# Clinical and genetic characteristics of amyotrophic lateral sclerosis patients with *ANXA11* variants

**DOI:** 10.1093/braincomms/fcac299

**Published:** 2022-11-16

**Authors:** Wonjae Sung, Minyeop Nahm, Su Min Lim, Min-Young Noh, Sanggon Lee, Sung-Min Hwang, Yong Ho Kim, Jinseok Park, Ki-Wook Oh, Chang-Seok Ki, Young-Eun Kim, Seung Hyun Kim

**Affiliations:** Department of Neurology, College of Medicine, Hanyang University, Seoul, Republic of Korea; Dementia Research Group, Korea Brain Research Institute, Daegu, Republic of Korea; Department of Neurology, College of Medicine, Hanyang University, Seoul, Republic of Korea; Department of Neurology, College of Medicine, Hanyang University, Seoul, Republic of Korea; Department of Neurology, College of Medicine, Hanyang University, Seoul, Republic of Korea; Gachon Pain Center and Department of Physiology, College of Medicine, Gachon University, Incheon, Republic of Korea; Gachon Pain Center and Department of Physiology, College of Medicine, Gachon University, Incheon, Republic of Korea; Department of Neurology, College of Medicine, Hanyang University, Seoul, Republic of Korea; Department of Neurology, College of Medicine, Hanyang University, Seoul, Republic of Korea; GC Genome, Yongin, Republic of Korea; Department of Laboratory Medicine, College of Medicine, Hanyang University, Seoul, Republic of Korea; Department of Neurology, College of Medicine, Hanyang University, Seoul, Republic of Korea

**Keywords:** amyotrophic lateral sclerosis, frontotemporal dementia, *ANXA11*, low-complexity domain, stress granule

## Abstract

Increasing genetic evidence supports the hypothesis that variants in the annexin A11 gene (*ANXA11*) contribute to amyotrophic lateral sclerosis pathogenesis. Therefore, we studied the clinical aspects of sporadic amyotrophic lateral sclerosis patients carrying *ANXA11* variants. We also implemented functional experiments to verify the pathogenicity of the hotspot variants associated with amyotrophic lateral sclerosis-frontotemporal dementia. Korean patients diagnosed with amyotrophic lateral sclerosis (*n* = 882) underwent genetic evaluations through next-generation sequencing, which identified 16 *ANXA11* variants in 26 patients. We analysed their clinical features, such as the age of onset, progression rate, initial symptoms and cognitive status. To evaluate the functional significance of the *ANXA11* variants in amyotrophic lateral sclerosis-frontotemporal dementia pathology, we additionally utilized patient fibroblasts carrying frontotemporal dementia-linked *ANXA11* variants (*p.P36R* and *p.D40G*) to perform a series of *in vitro* studies, including calcium imaging, stress granule dynamics and protein translation. The frequency of the pathogenic or likely pathogenic variants of *ANXA11* was 0.3% and the frequency of variants classified as variants of unknown significance was 2.6%. The patients with variants in the low-complexity domain presented unique clinical features, including late-onset, a high prevalence of amyotrophic lateral sclerosis-frontotemporal dementia, a fast initial progression rate and a high tendency for bulbar-onset compared with patients carrying variants in the C-terminal repeated annexin homology domains. In addition, functional studies using amyotrophic lateral sclerosis-frontotemporal dementia patient fibroblasts revealed that the *ANXA11* variants *p.P36R* and *p.D40G* impaired intracellular calcium homeostasis, stress granule disassembly and protein translation. This study suggests that the clinical manifestations of amyotrophic lateral sclerosis and amyotrophic lateral sclerosis-frontotemporal dementia spectrum patients with *ANXA11* variants could be distinctively characterized depending upon the location of the variant.

## Teaser

This study investigated the clinical aspects of sporadic amyotrophic lateral sclerosis patients carrying *ANXA11* variants and verified the pathogenicity of the hotspot variants associated with amyotrophic lateral sclerosis-frontotemporal dementia. Sung *et al*. reported unique clinical features of the patients with variants in the low-complexity domain with *in vitro* study results.

## Graphical Abstract

**Graphical abstract:**
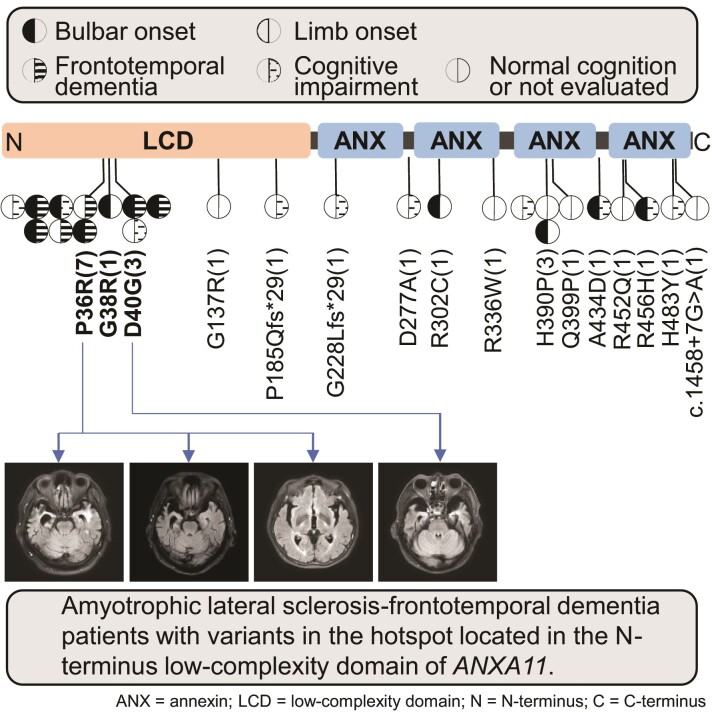

## Introduction

Amyotrophic lateral sclerosis (ALS) is a heterogeneous neurodegenerative disease presenting as progressive motor weakness due to motor neuron involvement in the motor cortex and spinal cord, associated with only 36–71 months of survival time.^1–3^ Notably, ALS also demonstrates non-motor manifestations ranging from mild cognitive impairment (CI) to severe frontotemporal dementia (FTD), resulting in behavioural, linguistic and executive dysfunctions.^4–7^ ALS and FTD causative genes harbouring low-complexity domains (LCDs) such as *TDP-43* and *FUS* are closely associated with stress granule (SG) dynamics.^8^ Variants in these genes perturb RNA metabolism and the disturbance causes the formation of cytoplasmic aggregates in neurons under disease conditions.^8^ Moreover, rapid advances in genetic screening, such as massively parallel sequencing in large cohorts, have made it possible to detect a variety of genetic variations, such as *C9orf72*, *TBK1*, *CHCHD10*, *MATR3* and *CCNF*, which cause ALS-FTD.^9–13^

Recently, there has been growing interest in the annexin A11 gene (*ANXA11*) as an ALS-linked gene. ANXA11 is a calcium-dependent phospholipid-binding protein and a member of the human annexin (ANX) protein family.^14^ It comprises an extended N-terminal domain, including a binding site for another calcium-binding protein, calcyclin and a C-terminal core containing four homologous ANX domains associated with phospholipid-binding via calcium regulation.^14,15^ Previous neuropathologic studies indicated that ALS-linked variants in the N-terminal LCD of *ANXA11*, *p.G38R* and *p.D40G*, caused cytoplasmic *ANXA11*-positive aggregates in patient motor neurons.^16,17^ In addition, the heterogeneous splice site variant c.1086 + 1G > A in the C-terminal ANX domain of *ANXA11* also induced the abnormal cytoplasmic accumulation of *ANXA11*.^18^ These findings imply that *ANXA11* variants associated with ALS have increased aggregation propensity, resulting in aberrant aggregation in the affected neurons. We recently reported that ALS-linked *ANXA11* variants identified from Korean sALS patients were associated with disturbed intracellular calcium homeostasis and SG dynamics.^19^ More specifically, N-terminal LCD variants with enhanced aggregation propensity. In contrast, C-terminal ANX domain variants affected the Ca^2+^-dependent nuclear membrane translocation of *ANXA11*.^19^ However, little is known about the relationship between domain-specific dysfunction and the clinical features of patients with *ANXA11* variants.

Recent genetic studies from independent ALS cohorts revealed that variants in *ANXA11* were responsible for both familial and sporadic ALS (sALS).^16–18,20–23^ These studies also demonstrated that several ALS patients carried *ANXA11* variants clustered in specific regions (hotspots) in the N-terminal LCD, such as *p.P36R*, *p.G38R* and *p.D40G*/*p.D40Y*.^16,17,20,23^ However, the detailed clinical characteristics of patients with *ANXA11* variants have not been analysed.

To link domain-specific *ANXA11* variants to clinical phenotypes, we expanded the exome data analysis of *ANXA11* in Korean sALS patients and dissected the distinctive clinical features of the patients with *ANXA11* variants. We also performed functional studies to address the pathogenic potential of the two hotspot variants, *p.P36R* and *p.D40G* using ALS-FTD patient cells.

## Materials and methods

### Participants and samples

All participants in this study were Korean and recruited from the ALS clinic of Hanyang University Hospital in Seoul, Korea, from November 2014 to December 2020. All patients met the diagnostic criteria for possible, probable, laboratory-supported or definite ALS according to the El Escorial Revised Criteria.^24^ To establish a more distinct disease entity, we excluded patients with diagnoses of a pure upper motor neuron phenotype or a pure lower motor neuron phenotype. After all participants signed written informed consent for genetic research, we collected peripheral blood samples from patients to screen for *ANXA11* variants and skin samples from patients carrying specific *ANXA11* variants and samples from healthy controls. The Institutional Review Board of Hanyang University Seoul Hospital approved the protocols for this study.

### Genetic analyses

Genomic DNA was extracted from peripheral blood leucocytes using a Wizard Genomic DNA Purification Kit according to the manufacturer’s instructions (Promega, Madison, WI, USA). We performed the whole-exome sequencing of 767 patients and the comprehensive multi-gene panel analysis of 115 patients. Sequencing libraries were prepared using either the TruSight™ one sequencing panel (Illumina Inc., San Diego, CA, USA) or the Agilent SureSelect All Exon 50 Mb kit (Agilent, Santa Clara, CA, USA) according to the manufacturer’s instructions. The flow cell was loaded onto either the MiSeq or the NextSeq 500 sequencing system (Illumina) for sequencing with 2 × 100 bp read lengths. The reads were mapped to the GRCh37/hg19 build using the Burrows–Wheeler Aligner and variants were called using GATK software. We filtered out all variants with allele frequencies of >0.01 based on various public databases, including the genome aggregation database (gnomAD, https://gnomad.broadinstitute.org) and the Korean Reference Genome Database (KRGDB, http://coda.nih.go.kr/coda/KRGDB/index.jsp). All identified variants were classified according to the American College of Medical Genetics and Genomics, the Association for Molecular Pathology guidelines^25^ and ClinGen recommendations (https://clinicalgenome.org/working-groups/sequence-variant-interpretation/). All likely pathogenic or pathogenic variants and variants of uncertain significance (VUS) were confirmed by DeepVariant (https://github.com/google/deepvariant) analysis or Sanger sequencing.

### Clinical data collection

We retrospectively collected information on ALS patients with *ANXA11* variants, including sex, age of onset, family history of ALS, site of the initial symptoms (spinal or bulbar), phenotype,^26^ the presence of other neurological manifestations such as FTD and multisystem proteinopathies (MSPs), neurophysiologic study results and revised ALS functional rating scale (ALSFRS-R) score by reviewing their medical records. The three-generational family histories of patients were obtained to determine whether they had sALS or familial ALS (fALS). In addition, we asked whether the patient’s family members had been diagnosed with ALS, other neurodegenerative diseases (such as Alzheimer’s disease and Parkinson’s disease), or MSPs (such as inclusion body myositis and Paget’s disease). Patients suspected of dominant or recessive inheritance with several affected family members were determined as fALS. In contrast, patients with a few family members diagnosed with neurodegenerative diseases without hereditary evidence were classified as having sALS. The ALSFRS-R contains a validated 12-item scale evaluating overall motor function, including bulbar and spinal (respiration and limbs) impairments.^27^ To determine whether there was a change in the speed at which the motor ability decreased in the early and intermediate stages of the disease, we calculated two types of progression rates, the early progression slope and the late progression slope, respectively. The early slope was the monthly decline in ALSFRS-R scores from the first symptom onset to the first consultation. As the mean follow-up duration of the subjects was about 12 months, researchers gathered additional ALSFRS-R scores obtained between 6 and 12 months after the initial evaluation and calculated the late slope. The formulas were as follows:

$$\begin{array}{rl} \mathrm{Early}\;\mathrm{slope}\;= & (48-\mathrm{ALSFRS}-R\;\mathrm{at}\;\mathrm{first}\;\mathrm{consultation})/\\ & \mathrm{months}\;\mathrm{from}\;\mathrm{symptom}\;\mathrm{to}\;\mathrm{the}\;\mathrm{first}\;\\ & \mathrm{consultation} \end{array}$$

$$\begin{array}{rl} \mathrm{Late}\;\mathrm{slope}\;= & (\mathrm{ALSFRS}\text{-}R\;\mathrm{at}\;\mathrm{first}\;\mathrm{consultation}\;\text{–}\mathrm{ALSFRS}\\ & -R\;\mathrm{score}\;\mathrm{at}\;\mathrm{follow}-\mathrm{up}\;\mathrm{period})/\\ & \mathrm{months}\;\mathrm{passed}\;\mathrm{since}\;\mathrm{the}\;\mathrm{first}\;\mathrm{visit} \end{array}$$

Furthermore, we collected patients’ survival data, such as the time to death, tracheostomy and permanent non-invasive positive pressure ventilation (>22 h daily for >7 days) according to Paganoni *et al*.^28^ to perform survival analysis. The events mentioned above were used as endpoints. In addition, individuals’ tracheostomy-free survival duration was defined as the period starting from symptom onset to the endpoint or the censoring date of 31 May 2021, for up to a maximum of 85 months. As some patients are not currently visiting our clinic, we additionally interviewed patients’ caregivers by telephone to figure out patients’ present status while reviewing their medical records related to survival.

We reviewed all patients’ T_2_-weighted, fluid-attenuated inversion recovery and T_1_-weighted images from brain MRIs. Experienced neuroradiologists confirmed the presence of temporal and frontal lobe atrophies and temporal horn dilatations, which were compatible with the FTD patients’ radiologic findings. They also excluded vascular lesions or severe white matter lesions, which could induce upper motor neuron signs.

The patients’ neuropsychological data were also evaluated to determine their behavioural and cognitive status. Most subjects had undergone the Seoul Neuropsychological Screening Battery (SNSB), one of Korea’s most commonly used neuropsychological tests.^29^ For patients who had not undergone the SNSB, we obtained scores on the Korean version of the Mini-Mental State Examination and the Frontal Assessment Battery.^30,31^ Based on the test results, the patients were classified into three subgroups. First, we categorized ALS patients with symptoms or test results suggesting FTD as having ALS-FTD. In detail, we sorted the ALS-FTD patients into three groups according to the results of the frontal/executive function test, language test and the presence of behavioural deterioration; behavioural variant FTD (bvFTD), semantic variant primary progressive aphasia (svPPA) and nonfluent/agrammatic variant primary progressive aphasia (nfavPPA).^32,33^ The second subgroup of patients, those with CI but not fulfilling the FTD criteria, were classified as having ALS with CI. Finally, the ALS-pure subgroup contained subjects without CI.^6,32,33^ Four patients had not performed any neuropsychological tests.

### *In vitro* functional studies

#### Cell culture and immunostaining

Adult human fibroblasts were extracted from the forearm skin by punch biopsy. Fibroblasts were cultured at 37% with 5% CO_2_ in media containing Dulbecco’s modified Eagles’ medium, non-essential amino acids (Gibco, Grand Island, NY, USA), sodium bicarbonate (Sigma-Aldrich) and 1% (vol/vol) penicillin/streptomycin/fungizone (Cellgro), supplemented with 20% heat-inactivated foetal bovine serum.

For immunostaining, cultured fibroblasts were fixed with 4% formaldehyde in phosphate-buffered saline (PBS) for 20 min at room temperature, permeabilized with 0.2% Triton X-100 in PBS for 15 min and blocked with 1% bovine serum albumin in PBS for 1 h. Then, the cells were incubated with mouse anti-G3BP antibody (1:1000, Millipore) overnight at 4% and labelled with fluorescein isothiocyanate-conjugated secondary antibodies (1:200, Jackson ImmunoResearch) for 60 min at room temperature. Images were acquired with a Leica TCS SP8 laser-scanning confocal microscope (Leica) using an HC PL APO CS2 63x/1.40 objective.

To quantify SG dynamics, we counted cells containing more than two puncta of G3BP1-positive granules in a randomly selected region (>50 fibroblasts). The experimental unit in this assay was individual fibroblast cultures. Results represent three independent fibroblast cultures (*n* = 3).

#### Measurement of intracellular calcium concentration and protein translation

To examine cytosolic calcium concentrations and protein translation, we performed Fura-2 calcium imaging and the surface sensing of translation (SUnSET) assay, respectively, as previously described.^19^ For measuring intracellular calcium concentration, fibroblasts were loaded with 2 µM Fura-2 AM (calcium-sensitive fluorescent ratiometric dye) for 40 min. Fluorescence ratios were measured with dual excitation wavelengths of 340 and 380 nm and an emission wavelength of 510 nm in response to calcium binding. Results are presented as the fluorescence ratio of fibroblasts excited at 340 nm relative to those excited at 380 nm. For the SUnSET assay, the intensity of puromycin incorporated protein (newly synthesized protein) was normalized to *GAPDH* protein level. The experimental unit in the western blot was each fibroblast culture. Results represent three independent fibroblast cultures (*n* = 3).

### Statistical analysis

Since we found different pathophysiology between amino-terminal variants within the LCD and carboxyl-terminal ANX domain variants in a previous study,^19^ we classified the subjects into two groups according to the location of the variant within *ANXA11.* Pearson’s χ^2^ test was used to compare categorical variables such as sex and onset site between the two groups. The Shapiro–Wilk test was applied to test the normality of the data distribution. Continuous variables such as the age of onset and progression rates were analysed using the two-sample *T*-test and the exact Wilcoxon rank-sum test. We also applied the Cochran–Armitage test to evaluate the tendency towards cognitive decline in both groups. The Kaplan–Meier survival analysis and the log-rank test were used to compare the tracheostomy-free survival periods of the two groups. In *in vitro* studies, comparisons were made by one-way ANOVA analysis with *post hoc* Tukey tests using GraphPad Prism 7. The cut-off for significance was set at *P*-values of <0.05. We carried out statistical analyses with IBM SPSS Statistics for Windows 26.0 (Chicago, IL, USA) and R software (version 3.6.0.).

### Data availability

The authors confirm that the data supporting the findings of this study are available within the article and its Supplementary material.

## Results

### Genetic analysis of *ANXA11* variants in ALS patients

**Figure 1:**
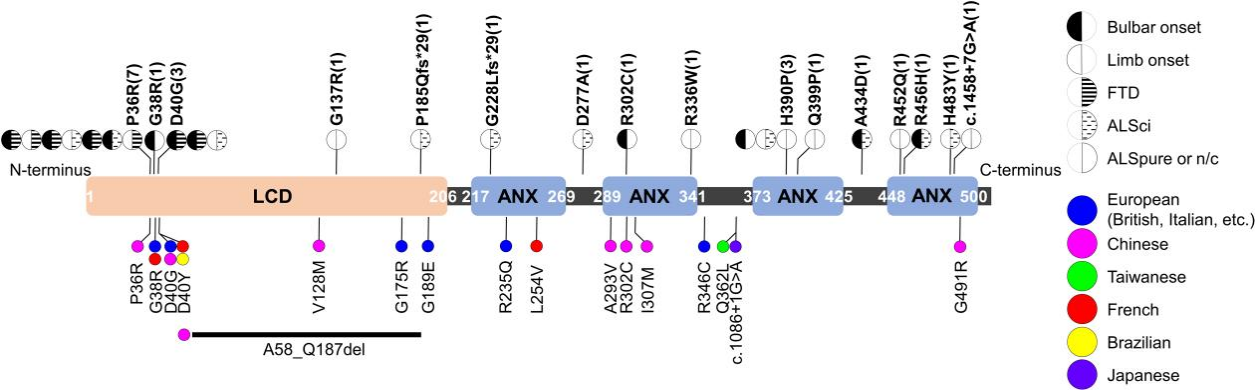
**Schematics of the protein domain structure of ANXA11 and identified variants.** The upward lollipops indicate variants detected in this study. Each subject’s clinical characteristics, such as the site of onset and cognitive status, are depicted by distinct patterns. The numbers in parenthesis refer to the number of patients with the corresponding variant. The downward lollipops represent variants identified in other studies. Variants found in the patients of each country are expressed in different colours. SMART (simple modular architecture research tool—http://smart.embl-heidelberg.de) was used to predict the domains. n/c, not checkable.

We previously identified nine *ANXA11* variants in 13 patients through the exome sequence analysis of 500 Korean sALS patients and found that the LCD (N-terminus of *ANXA11*) variants *p.G38R* and *p.D40G* increased the propensity for aggregation, whereas the ANX domain (C-terminus of *ANXA11*) variants *p.H390P* and *p.R456H* altered Ca^2+^ responses.^19^ We expanded our genetic study to discriminate the clinical features presented by the domain-specific variants. We recruited 26 patients with 16 different *ANXA11* variants. All the subjects carrying *ANXA11* variants were Korean sALS patients. The three patients carried *p.D40G*, which was classified as a likely pathogenic variant (3/882, 0.3%). In addition, 15 VUS from 23 patients were identified (23/882, 2.6%); *p.P36R* in 7 patients, *p.H390P* in 3 patients and *p.G38R*, *p.G137R*, p.P185Qfs*29, p.G228Lfs*29, *p.D277A*, *p.R302C*, *p.R336W*, *p.Q399P*, *p.A434D*, *p.R452Q*, *p.R456H*, *p.H483Y* and c.1458 + 7G > A, respectively, in 9 different patients (Fig. 1, Supplementary Table 1). The 12 patients had missense variants in the N-terminal LCD of *ANXA11* and 1 patient showed a frameshift variant in this domain. The variants of 11 patients were clustered at amino acids 36, 38 and 40. The seven patients had *p.P36R*, one subject carried *p.G38R* and three patients showed the *p.D40G* variant. We also found 9 missense variants, 1 frameshift variant and 1 splicing variant in 13 patients with variants in the C-terminal ANX domain.

### Clinical characteristics of patients carrying *ANXA11* variants

**Figure 2:**
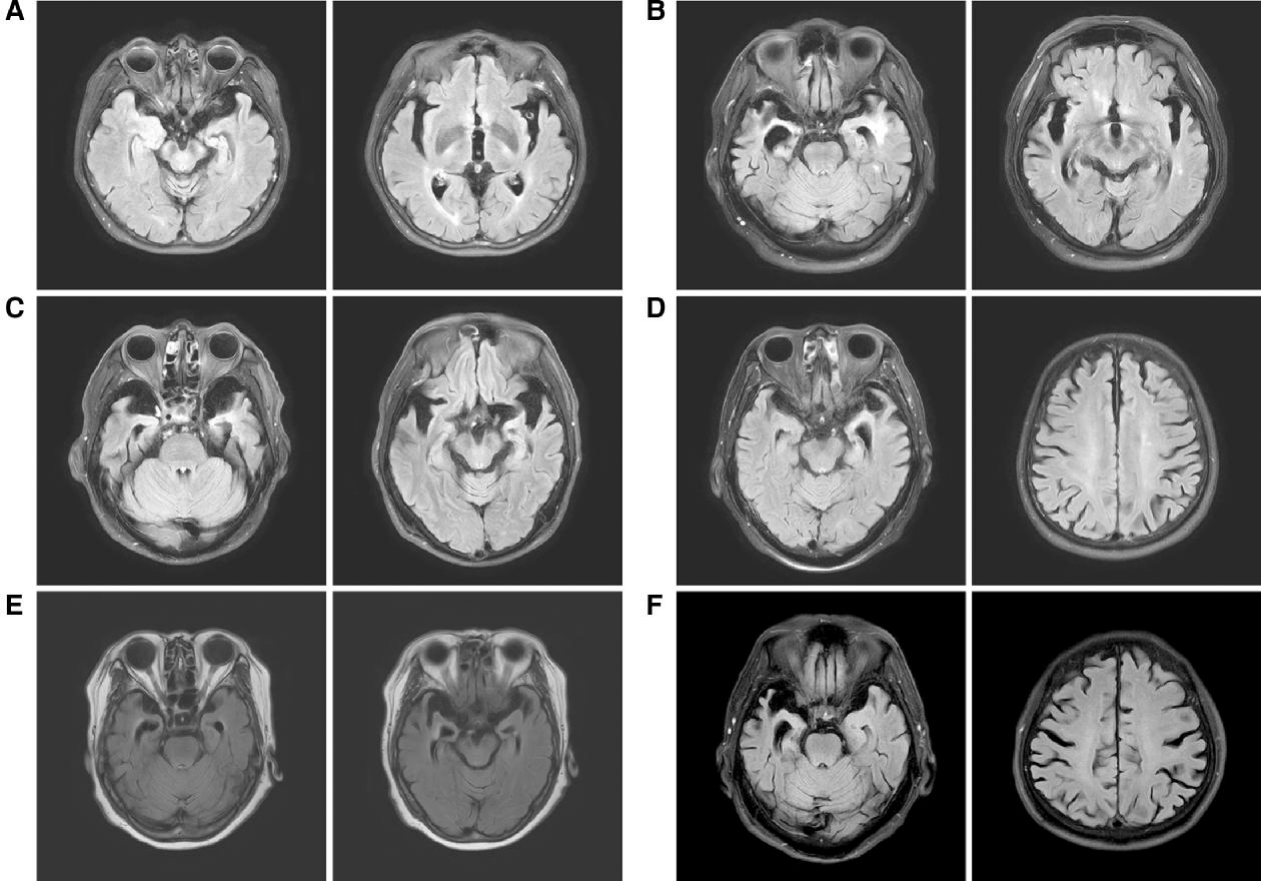
**T_2_-weighted fluid-attenuated inversion recovery axial brain MRIs of ALS-FTD patients.** (**A–C**) Images demonstrate bilateral temporal atrophy combined with dilatation of both temporal horns. (**D**) Left-dominant temporal atrophy with diffuse brain atrophy was present in an nfavPPA patient. (**E** and (**F**) Images present disproportionate atrophy of the bilateral temporal lobes, which is worse on the right. Both patients were diagnosed with the behavioural variant of FTD.

Next, we compared the clinical characteristics of the patients in the LCD variant and other ANX domain variant groups. Baseline demographic and disease characteristics are summarized in Table 1 and Supplementary Table 2. The sex ratio did not differ significantly between the two groups. The age of onset was older in the LCD variant group than in the ANX domain variant group, showing statistical significance (*P* = 0.002). The early slope was significantly faster in the LCD variant group (*P* = 0.026). Although the late slope was not statistically different (*P* = 0.162), the slope was steeper in the LCD variant group. Most subjects carrying variants in the LCD region experienced bulbar problems (dysarthria or dysphagia) as the first symptom of ALS. The bulbar-onset to-limb onset ratio was higher in the LCD variant group, but there was no significant difference between the two groups (*P* = 0.23). Similarly, the bulbar phenotype was the most common in the LCD variant group (*n* = 7, 53.8%), whereas the classical phenotype was the most common in the ANX domain variant group (*n* = 7, 53.8%). All electromyography data from patients showed neurogenic patterns. None of the subjects demonstrated the coexistence of diseases involving muscle or bone. We found a higher risk of CI in the LCD variant group (*P* < 0.05). While there were no ALS-FTD patients in the ANX domain variant group, seven subjects were diagnosed with ALS-FTD in the LCD variant group. The three patients were diagnosed with probable bvFTD. In addition, two patients were concluded to have imaging-supported svPPA and two patients had imaging-supported nfavPPA according to the previously presented diagnostic criteria.^33^ All of these patients diagnosed with ALS-FTD showed inherent atrophy of the frontal and temporal lobes, either bilaterally or disproportionately (Fig. 2). The five of them had the *p.P36R* variant and the other two patients carried the *p.D40G* variant. In contrast, no patient harbouring an ANX domain variant presented with severe cognitive decline.

**Table 1:** Comparison of demographic and clinical features between groups classified according to the location of *ANXA11* variants

	LCD variant group (*n* = 13)	ANX domain variant group (*n* = 13)	*P*-value
Sex, *n* (%)	–	–	0.69^a^
Male	7 (53.8)	8 (61.5)	
Female	6 (46.2)	5 (38.5)	
Age of onset	–	–	**0**.**002**^b^
Mean ± SD	69.3 ± 7.8	59 ± 7.5	
Early slope^c^	–	–	**0**.**026**^d^
Median (range)	1.20 (1.00–1.88)	0.77 (0.32–0.90)	
Late slope^e^	–	–	0.162^b^
Mean ± SD	1.63 ± 0.83	1.14 ± 0.91	
Site of onset, *n* (%)	–	–	0.23^a^
Bulbar	7 (53.8)	4 (30.7)	
Limb	6 (46.2)	9 (69.3)	
Classification by cognition, *n* (%)	–	–	**<0**.**05**^f^
ALS-FTD	7 (53.8)	0 (0)	
ALSci	4 (30.8)	6 (46.2)	
ALS-pure	0 (0)	6 (46.2)	
Not checked	2 (15.4)	1 (7.6)	

Bold numbers indicate *P*-values <0.05. *ANXA11*, annexin A11; LCD, low-complexity domain; ALS-FTD, amyotrophic lateral sclerosis-frontotemporal dementia; ALSci, amyotrophic lateral sclerosis with cognitive impairment; ALS-pure, amyotrophic lateral sclerosis-pure (without cognitive impairment); svPPA, semantic variant primary progressive aphasia; nfavPPA, nonfluent/agrammatic variant; SD, standard deviation.

^a^ Pearson’s χ^2^ test for sex and site of onset variables between the LCD and ANX groups.

^b^ Independent two-sample *t*-test for the age of onset and late slope variable between the LCD domain and ANX groups after normality checking with the Shapiro–Wilk test.

^c^ Early slope = (48—ALSFRS-R at first consultation)/months from symptom onset to the first consultation.

^d^ The exact Wilcoxon rank-sum test was used to evaluate the early slope.

^e^ Late slope = (ALSFRS-R at first consultation—ALSFRS-R score at follow-up period)/months passed since the first visit.

^f^ The Cochran–Armitage test was used to determine the tendency towards a cognitive decline in both groups.

In the survival analysis, nine patients carrying LCD variants and seven patients with ANX domain variants met the endpoint criteria during the follow-up period. The median survival duration was 45.0 (24.0–57.0) months for the group with LCD variants and 34.0 (27.0–38.0) months for the group with ANX domain variants (Supplementary Table 3). The 5-year survival rate was 10.2% for the group with LCD variants and 36.7% for the group with ANX domain variants. However, the Kaplan–Meier survival analysis showed no significant difference in survival rates between the two groups (*P* = 0.72; Supplementary Fig. 1).

### *In vitro* functional studies

#### ALS-FTD-linked variants of *ANXA11* cause impaired intracellular calcium homeostasis

**Figure 3:**
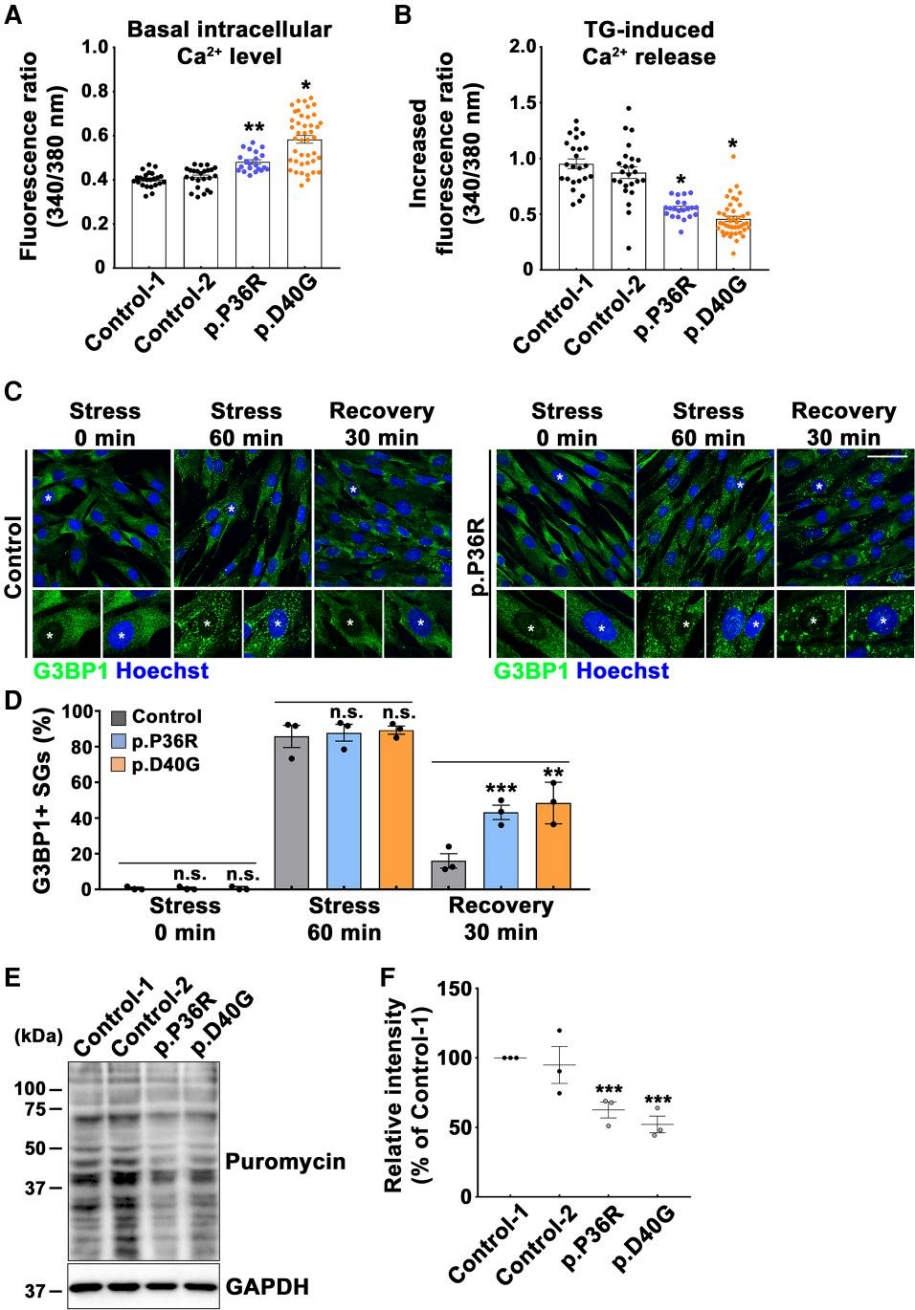
**p.P36R and p.D40G variants in *ANXA11* reveal disruption of intracellular Ca^2+^ homeostasis and abnormal SG disassembly along with impaired global protein synthesis.** (**A**, **B**) The Ca^2+^ concentration in each set of cultured fibroblasts was analysed via Fura-2 calcium imaging. (**A**) The basal cytosolic Ca^2+^ levels of healthy control fibroblasts and patient fibroblasts (Controls 1 and 2, *n* = 24; *p.P36R*, *n* = 21; *p.D40G*, *n* = 43). (**B**) Released Ca^2+^ concentration from the endoplasmic reticulum in controls and patient fibroblasts after stimulating cells with thapsigargin (Controls 1 and 2, *n* = 24; *p.P36R*, *n* = 21; *p.D40G*, *n* = 43). (**C**, **D**) Cultured fibroblasts of healthy control and ALS-FTD patients were treated with sodium arsenite to provoke SG aggregation. After reaching the assembly phase, sodium arsenite was replaced with fresh culture medium to disassemble SGs. (**C**) *G3BP1*-positive SGs in the presence of oxidative stress and at the recovery phase without sodium arsenite were visualized by immunofluorescence. The scale bar represents 50 μm. (**D**) The percentage of *G3BP1*-positive SGs during the assembly and disassembly phases was determined and shown in the graph. To quantify stress granules dynamics, we counted the cells that contained more than two *G3BP1*-positive granules per cell (>50 cells, *n* = 3). (**E**, **F**) Global translation activities of fibroblasts were analysed by the SUnSET assay. (**E**) Proteins synthesized in the fibroblasts of controls and patients were probed by immunoblotting using anti-puromycin and anti-*GAPDH* antibodies. (**F**) Quantification of puromycin levels normalized to *GAPDH* levels from three independent experiments by densitometry. The values are the mean ± SEM. All comparisons were made against Control 1. *P < 0.001, **P < 0.01, ***P < 0.05. ns, not significant; one-way ANOVA with *post hoc* Tukey tests.

Since *ANXA11* variants are linked to an ALS-FTD cluster within the N-terminal LCD, we performed a series of functional studies on the *p.P36R* and *p.D40G* variants using patient fibroblasts to understand the contribution of *ANXA11* variants to ALS-FTD pathology. First, to explore if the ALS-FTD-linked *ANXA11* variants expressed defective Ca^2+^ signalling, we used Fura-2 ratio calcium imaging to quantify intracellular Ca^2+^ levels in passage-matched patient fibroblasts carrying the *p.P36R* or *p.D40G* variant. The two healthy control fibroblast cultures had similar cytoplasmic Ca^2+^ levels (Fig. 3A, Control 1, a 43-year-old male; Control 2, a 57-year-old female). However, the fibroblasts from two ALS-FTD patients carrying the *p.P36R* or *p.D40G* variant had significantly higher basal cytosolic Ca^2+^ concentrations than healthy control fibroblasts (Fig. 3A). Next, we measured endoplasmic reticulum (ER) Ca^2+^ content by stimulating the fibroblasts with the sarco/endoplasmic reticulum Ca^2+^-ATPase inhibitor thapsigargin. Patient fibroblasts carrying the ALS-FTD-linked variants of *ANXA11* showed considerably lower thapsigargin evoked Ca^2+^ release than the healthy control fibroblasts, indicating lower ER Ca^2+^ levels (Fig. 3B). These findings imply that *ANXA11* variants linked to ALS-FTD might disrupt intracellular Ca^2+^ homeostasis.

#### ALS-FTD-linked variants of *ANXA11* cause aberrant SG dynamics and global translation

To investigate whether SG dynamics were affected by ALS-FTD variants of *ANXA11*, we cultured ALS-FTD patient fibroblasts carrying the *p.P36R* or *p.D40G* variant in the presence of sodium arsenite, an oxidative stress inducer, to promote SG assembly. Then, we converted SG dynamics to the disassembly phase by replacing sodium arsenite with fresh culture medium. After treatment with 0.5 mM sodium arsenite for 60 min, *G3BP1*-positive SGs were observed in the fibroblasts of both the controls and ALS-FTD patients (Fig. 3C and D). In contrast, fibroblasts from two ALS-FTD patients displayed significantly prolonged SG disassembly. After resting for 30 min with fresh media, only about 16% of the assembled SGs remained in the control fibroblasts, whereas >43 and 48% of the SGs failed to disassemble in patient fibroblasts carrying the *p.P36R* or *p.D40G* variant, respectively (Fig. 3C and D). Furthermore, we obtained similar results using neuron-like cells. In NSC-34 motor neuron-like cells expressing C-terminal GFP-tagged *ANXA11* wild type (WT), *P36R* and *D40G* variants, SG assembly was induced by treatment with 0.5 mM sodium arsenite for 30 min. We then replaced the stressor with fresh culture media for 30 min to initiate the disassembly process. Under the basal condition, no SGs were observed in any experimental group, suggesting that overexpression of *ANXA11* constructs did not affect SG assembly. However, treatment with sodium arsenite strongly induced *G3BP1-*positive SGs formation, which colocalized with the WT or LCD mutations of *ANXA11* in a similar manner. Consistent with results using fibroblasts, cells expressing LCD variants of *ANXA11* resulted in significantly prolonged SG disassembly compared with WT (Supplementary Fig. 2). We also performed a SUnSET assay to evaluate global translation activity in patient fibroblasts carrying the *p.P36R* or *p.D40G* variant of *ANXA11*. We found that protein synthesis was significantly decreased in patient fibroblasts compared with healthy controls (Fig. 3E and F). Our results suggest that the ALS-FTD-linked LCD variants of *ANXA11* altered SG disassembly and impaired global translation.

## Discussion

Our studies revealed two distinctive characteristics supporting the genetic and clinical contribution of *ANXA11* variants in ALS. First, *ANXA11* might be listed as one of the most common genes with variants in Asian sALS patients. We identified 16 different variants of *ANXA11* in 26 patients from 882 Korean sALS subjects. Among 26 patients, three were classified as carrying pathogenic or likely pathogenic variants, accounting for a variant frequency of 0.3% (three *p.D40G* patients). VUS were identified in 2.6% of the sALS patients overall (23 patients). The total frequency of *ANXA11* variants, including VUS, was 2.9%.

Previous studies from various countries have reported numerous variants in *ANXA11* and variant frequencies in each ethnic group of ALS patients. Smith *et al*.^16^ performed whole-exome sequencing in 751 European fALS and 180 sALS subjects and identified six *ANXA11* variants in 12 ALS patients. Moreover, three *ANXA11* variants in five patients were detected among 150 fALS and 180 sALS from the French ALS cohort.^17^ The variant frequencies of *ANXA11* investigated in Europeans accounted for 1.3% in fALS and 1.4% in sALS. Research in Asian countries has investigated *ANXA11* variants in their ALS patients. Zhang *et al*.^20^ showed six nonsynonymous heterozygous *ANXA11* variants in nine patients, accounting for a variant frequency of 2.3% in Chinese sALS patients. Further research on the Chinese and Taiwanese ALS patients identified three novel missense variants and one splicing variant detected in Japanese patients was reported as a post-mortem tissue neuropathologic finding.^18,21,22^ The same variant, *p.D40Y*, previously reported in French ALS patients, was also identified in the two Brazilian fALS patients.^23^ Our results are closer to those found in the Chinese patients with ALS.^20^ The frequency of *ANXA11* variants in our data and the Chinese cohort was relatively higher than that of the European cohort. In addition, no fALS patients harbouring *ANXA11* variants were found. Ethnic differences might have been responsible for these findings. A previous study reporting a low rate of fALS in Koreans compared with Europeans supports this hypothesis (Supplementary Table 3).^3,34^ The *ANXA11* variant frequency was higher than previously known ALS-related genes because the *ANXA11* is relatively longer, which can allow more VUS.

Second, we identified distinctive clinical features of ALS patients carrying *ANXA11* variants. We recently determined that the N-terminus LCD variants *p.G38R* and *p.D40G* increased aggregation propensity, whereas the C-terminus ANX domain variants p.H390P and *p.R456H* altered Ca^2+^ responses.^19^ Based on these findings, we hypothesized that different pathomechanisms could result in distinctive clinical features. Therefore, we classified the subjects into two groups, LCD and ANX domain variants, rather than independent variants, to compare the clinical characteristics of the two groups. Thus, it was possible to identify significant differences in the clinical characteristics between the two groups, which have not been previously reported. A previous study reported the old onset age (average, 67 years) of ALS patients carrying *ANXA11* variants.^16^ All subjects included in the current study also showed a late age of onset (average, 64.2) compared with the average age of onset in the Korean ALS patients (average, 60.4; Supplementary Table 3).^3^ In patients with *ANXA11* variants, the age of onset was significantly older in patients carrying LCD variants than in patients with ANX domain variants. Notably, about half of the patients harbouring variants located at the N-terminal LCD were diagnosed with ALS-FTD. The variants were found in residues 36 and 40 (*p.P36R* and *p.D40G*). Considering the low prevalence of ALS-FTD (4.8%) in our previous sALS cohort study, we regard this finding of numerous ALS-FTD patients carrying variants clustered in a specific region of a single gene as a highly significant discovery (Supplementary Table 3).^35^ In addition, patients with variants in the *ANXA11* LCD tended to experience bulbar problems as the first symptom of ALS in contrast to patients carrying ANX domain variants, although there was no statistical significance. However, it was still possible to identify a higher incidence of bulbar-onset ALS patients in those with *ANXA11* variants than in those without *ANXA11* variants (Supplementary Table 3).^36^ In terms of the progression speed, a steeper deterioration rate in the early stage of the disease was detected in patients with LCD variants than in patients carrying ANX domain variants. Unfortunately, no apparent cause for these differences has been identified. We previously reported experimental results showing that missense variants in both the LCD and ANX domains caused abnormal liquid–liquid phase separation and altered SG dynamics, indicating the possibility of cytosolic aggregate formation in the affected neurons. Thus, a comparative analysis of each aggregate interactome induced by domain-specific variants may be helpful for understanding genotype–phenotype correlations. More extensive research, such as expanding the number of patient observations and detailed functional studies of each variant, is needed to pinpoint the clinical features of ALS patients associated with *ANXA11* variants.

Meanwhile, we could not detect patients additionally diagnosed with other systemic-involving diseases such as inclusion body myopathy or Paget’s disease of bones. There are several potential causes for this result. Although the location of the missense variant was identical between this study and the prior research by Leoni *et al*.,^23^ produced amino acids were distinct since replaced nucleic acids were different. Moreover, identifying the various phenotypes caused by *ANXA11* variants is elusive in a situation where only one family member is affected without a family history. From this perspective, these differences might affect the manifestation of the disease.

Increasing evidence suggests that ALS and FTD share genetic risk factors and pathological features displaying intracellular protein aggregates. Our genetic data revealed that most *ANXA11* variants were linked to the ALS-FTD cluster within the N-terminal domain, which corresponds to an LCD responsible for aggregation. Importantly, the post-mortem tissue of the patients with *p.G38R* and *p.D40G* variants presented abundant ANXA11-positive aggregates within the motor neurons and neurons in the neocortex.^16,17^ Furthermore, variants in the specific genes linked to ALS-FTD such as *TDP-43* and *FUS* interrupted the reversible liquid–liquid phase separation of the coding proteins, eventually promoting pathological accumulation.^37^ We previously demonstrated that *ANXA11*, *G38R* and *D40G* recombinant proteins enhanced protein aggregation propensity by inducing irreversible liquid–liquid phase separation.^19^ Moreover, in the present study using patient fibroblasts, we performed functional studies of the *p.P36R* and *p.D40G* variants to determine whether they played a role in ALS-FTD. Patient fibroblasts carrying ALS-FTD-linked *ANXA11* variants showed functional defects related to intracellular calcium homeostasis, SGs dynamics and protein translation. Consequently, considering all of the molecular mechanism results, variants in Residues 36 and 40 in *ANXA11* seem to be strongly linked to ALS and ALS-FTD pathogenesis.

There were some limitations in this study. First, the clinical significance of most of the variants identified in this study has not yet been determined. Some allele frequencies of *ANXA11* variants in the ANX domain were relatively higher in the total and non-neuro East Asian cohorts than in the entire cohort. Nonetheless, their frequencies are not sufficient to classify them as benign variants. Moreover, our previous study has revealed that the carboxyl-terminal ANX domain variants *p.H390P* and *p.R456H* can alter Ca^2+^ and contribute to ALS pathogenesis.^19^ These findings suggest that variants in the ANX domain should be categorized as VUS variants. Insufficient information on the causality between variants and pathogenicity still exists. However, replicated genetic, neuropathological and functional studies have established *ANXA11* as an ALS-associated gene and proved the pathogenicity of *ANXA11* variants.^16–22^ Hence, we speculate that more variants of *ANXA11* will be confirmed as the gene has been solidly linked to ALS. Additional data, such as segregation data within families or parental genotyping and further studies identifying the pathogenicity of each variant will be helpful for establishing the direct linkage between ALS and variants. Second, we addressed the pathogenic potential of two ALS-FTD-linked variants using patient skin fibroblasts. Although our data clearly showed functional defects in the *ANXA11* missense variants, the findings should be verified in disease-relevant cell models such as induced pluripotent stem cell-derived motor neurons. Variants identified as VUS located in the ANX domain should also be experimentally characterized to understand the contribution of these variants to diseases.

Despite these limitations, our study supported *ANXA11* as an ALS and ALS-FTD gene and revealed distinctive clinical characteristics of ALS patients carrying domain-specific variants. We detected many ALS patients with the *p.P36R* variant and observed functional defects that could alter intracellular calcium homeostasis and SG dynamics, suggesting the *p.P36R* variant of *ANXA11* as a variant strongly linked to ALS pathogenesis. This study also indicated that the variants in the hotspot regions (amino acid Residues 36 and 40) located in the N-terminus LCD of *ANXA11* were highly associated with ALS-FTD.

## Supplementary Material

fcac299_Supplementary_Data

## Abbreviations

ALS =: amyotrophic lateral sclerosis
ALSFRS-R =: revised amyotrophic lateral sclerosis functional rating scale
ALS-FTD =: amyotrophic lateral sclerosis-frontotemporal dementia
ANX =: annexin
ANXA11 =: annexin A11
bvFTD =: behavioural variant frontotemporal dementia
fALS =: familial amyotrophic lateral sclerosis
FTD =: frontotemporal dementia
LCD =: low-complexity domain
MSP =: multisystem proteinopathies
nfavPPA =: nonfluent/agrammatic variant primary progressive aphasia
sALS =: sporadic amyotrophic lateral sclerosis
SG =: stress granule
SNSB =: Seoul Neuropsychological Screening Battery
svPPA =: semantic variant primary progressive aphasia
VUS =: variants of uncertain significance
WT =: wild type
