# Supplementary material for: Clinical and genetic characteristics of amyotrophic lateral sclerosis patients with ANXA11 variants
Source: Brain Commun. 2022 Nov 16;4(6):fcac299. doi: 10.1093/braincomms/fcac299 (PMC9707645; doi:10.1093/braincomms/fcac299)
Supplement: fcac299_Supplementary_Data [file fcac299_supplementary_data.pdf]

Supplementary Table 1. Genetic details of *ANXA11* variants in Korean ALS patients

MND	Source	Protein	cDNA	Variant classification	dbSNP147	gnomAD_total	gnomAD_EAS	gnomAD_total (non-neuro)	gnomAD_EAS (non-neuro)	SIFT _pred:score	MutationTaster_ pred:score	FATHMM_ pred:score
1876	sALS	NP_001148.1:p.P36R	NM_001157.2:c.107C>G	VUS	rs199988035	N/A	N/A	N/A	N/A	D:0	D:1	T:4.49
1742	sALS	NP_001148.1:p.P36R	NM_001157.2:c.107C>G	VUS	rs199988035	N/A	N/A	N/A	N/A	D:0	D:1	T:4.49
2011	sALS	NP_001148.1:p.P36R	NM_001157.2:c.107C>G	VUS	rs199988035	N/A	N/A	N/A	N/A	D:0	D:1	T:4.49
2516	sALS	NP_001148.1:p.P36R	NM_001157.2:c.107C>G	VUS	rs199988035	N/A	N/A	N/A	N/A	D:0	D:1	T:4.49
2807	sALS	NP_001148.1:p.P36R	NM_001157.2:c.107C>G	VUS	rs199988035	N/A	N/A	N/A	N/A	D:0	D:1	T:4.49
2885	sALS	NP_001148.1:p.P36R	NM_001157.2:c.107C>G	VUS	rs199988035	N/A	N/A	N/A	N/A	D:0	D:1	T:4.49
2925	sALS	NP_001148.1:p.P36R	NM_001157.2:c.107C>G	VUS	rs199988035	N/A	N/A	N/A	N/A	D:0	D:1	T:4.49
1656	sALS	NP_001148.1:p.G38R	NM_001157.2:c.112G>A	VUS	rs142083484	0.00004444	0	0.00005303	0	D:0	D:1	T:4.33
2256	sALS	NP_001148.1:p.D40G	NM_001157.2:c.119A>G	LPV	rs1247392012	0.00001166	0	N/A	N/A	T:0.49	D:0.999	T:4.57
1818	sALS	NP_001148.1:p.D40G	NM_001157.2:c.119A>G	LPV	rs1247392012	0.00001166	0	N/A	N/A	T:0.49	D:0.999	T:4.57
2660	sALS	NP_001148.1:p.D40G	NM_001157.2:c.119A>G	LPV	rs1247392012	0.00001166	0	N/A	N/A	T:0.49	D:0.999	T:4.57
1761	sALS	NP_001148.1:p.G137R	NM_001157.2:c.409G>A	VUS	rs530828539	0.00004053	0	0.00004856	0	D:0.02	D:0.999	T:4.38
2303	sALS	NP_001148.1:p.P185Qfs*29	NM_001157.2:c.554del	VUS	N/A	N/A	N/A	N/A	N/A	N/A	N/A	N/A
861	sALS	NP_001148.1:p.G228Lfs*29	NM_001157.2:c.682_686delins TTG	VUS	N/A	N/A	N/A	N/A	N/A	N/A	N/A	N/A
1943	sALS	NP_001148.1:p.D277A	NM_001157.2:c.830A>C	VUS	N/A	N/A	N/A	N/A	N/A	T:0.07	D:1	T:3.38
2474	sALS	NP_001148.1:p.R302C	NM_001157.2:c.904C>T	VUS	rs142183550	0.00004874	0.0004	0.00005286	0.0004472	D:0	D:1	T:2.64
2350	sALS	NP_001148.1:p.R336W	NM_001157.2:c.1006C>T	VUS	rs368700483	0.00003299	0.0002	0.00003347	0.00008030	D:0	D:1	T:3.82
1653	sALS	NP_001148.1:p.H390P	NM_001157.2:c.1169A>C	VUS	rs749573800	0.000008173	0.0001	0.000009679	0.0001495	D:0.01	D:1	T:3.77
2103	sALS	NP_001148.1:p.H390P	NM_001157.2:c.1169A>C	VUS	rs749573800	0.000008173	0.0001	0.000009679	0.0001495	D:0.01	D:1	T:3.77
2437	sALS	NP_001148.1:p.H390P	NM_001157.2:c.1169A>C	VUS	rs749573800	0.000008173	0.0001	0.000009679	0.0001495	D:0.01	D:1	T:3.77
2518	sALS	NP_001148.1:p.Q399P	NM_001157.2:c.1196A>C	VUS	rs1589414993	N/A	N/A	N/A	N/A	D:0.03	D:1	T:3.81
2725	sALS	NP_001148.1:p.A434D	NM_001157.2:c.1301C>A	VUS	rs1293056598	N/A	N/A	N/A	N/A	D:0	D:1	T:3.44
2344	sALS	NP_001148.1:p.R452Q	NM_001157.2:c.1355G>A	VUS	rs142001721	0.00003254	0.00005798	0.00004331	0.0001490	T:0.17	D:0.986	T:4.03
1802	sALS	NP_001148.1:p.R456H	NM_001157.2:c.1367G>A	VUS	rs749809198	0.00004471	N/A	0.00005672	0	D:0	D:1	T:2.97
2722	sALS	NP_001148.1:p.H483Y	NM_001157.2:c.1447C>T	VUS	N/A	N/A	N/A	N/A	N/A	D:0.04	P:0.76	T:3.93
1522	sALS	-	NM_001157.2:c.1458+7G>A	VUS	rs755226655	0.00006018	0.0008160	0.00005811	0.0008946	N/A	N/A	N/A

Supplementary Table 1. Continued

MND	FATHMM-mkl _pred:score	VEST3_score	MutationAssessor _pred:score	CADD_Phred	Provean _pred:score	PP2_HDIV _pred:score	PP2_HVAR _pred:score	LRT_pred:score	DANN_score	PhastCons20way _mammalian	GERPrs_score	SiPhy_29way _logOdds	PhyloP20way _mammalian
1876	D:0.924	0.612	M:2.445	13.15	N:-1.76	D:0.998	D:0.987	N:0.896	0.972	0.987	4.7	13.517	1.036
1742	D:0.924	0.612	M:2.445	13.15	N:-1.76	D:0.998	D:0.987	N:0.896	0.972	0.987	4.7	13.517	1.036
2011	D:0.924	0.612	M:2.445	13.15	N:-1.76	D:0.998	D:0.987	N:0.896	0.972	0.987	4.7	13.517	1.036
2516	D:0.924	0.612	M:2.445	13.15	N:-1.76	D:0.998	D:0.987	N:0.896	0.972	0.987	4.7	13.517	1.036
2807	D:0.924	0.612	M:2.445	13.15	N:-1.76	D:0.998	D:0.987	N:0.896	0.972	0.987	4.7	13.517	1.036
2885	D:0.924	0.612	M:2.445	13.15	N:-1.76	D:0.998	D:0.987	N:0.896	0.972	0.987	4.7	13.517	1.036
2925	D:0.924	0.612	M:2.445	13.15	N:-1.76	D:0.998	D:0.987	N:0.896	0.972	0.987	4.7	13.517	1.036
1656	D:0.878	0.731	M:2.675	23.5	N:-1.6	D:1	D:0.926	N:0.006	0.996	0.99	3.78	12.263	0.925
2256	D:0.893	0.625	L:1.18	14.13	N:-0.24	D:0.97	P:0.587	N:0.632	0.98	0.986	4.69	12.418	1.049
1818	D:0.893	0.625	L:1.18	14.13	N:-0.24	D:0.97	P:0.587	N:0.632	0.98	0.986	4.69	12.418	1.049
2660	D:0.893	0.625	L:1.18	14.13	N:-0.24	D:0.97	P:0.587	N:0.632	0.98	0.986	4.69	12.418	1.049
1761	N:0.31	0.57	M:2.795	23.7	N:-1.95	D:1	D:0.946	N:0.184	0.996	0.098	5.45	17.169	0.935
2303	N/A	N/A	N/A	N/A	N/A	N/A	N/A	N/A	N/A	N/A	N/A	N/A	N/A
861	N/A	N/A	N/A	N/A	N/A	N/A	N/A	N/A	N/A	N/A	N/A	N/A	N/A
1943	D:0.914	0.862	M:2.9	25.2	D:-6.73	D:0.991	D:0.915	D:0	0.995	0.686	5.6	14.038	0.993
2474	D:0.766	0.975	M:3.42	34	D:-7	D:1	D:1	D:0	0.999	0.876	3.6	7.057	0.079
2350	D:0.766	0.943	H:3.925	32	D:-7.28	D:1	D:0.997	D:0	0.999	0.996	-1.15	14.913	0.099
1653	D:0.99	0.666	H:3.63	29.5	D:-7.1	D:1	D:1	D:0	0.988	1	5.07	13.097	1.061
2103	D:0.99	0.666	H:3.63	29.5	D:-7.1	D:1	D:1	D:0	0.988	1	5.07	13.097	1.061
2437	D:0.99	0.666	H:3.63	29.5	D:-7.1	D:1	D:1	D:0	0.988	1	5.07	13.097	1.061
2518	D:0.875	0.548	L:1.7	29	D:-2.71	D:1	D:1	D:0	0.996	0.998	5.14	13.226	1.061
2725	D:0.89	0.512	M:2.225	32	D:-3.86	D:0.992	P:0.672	D:0	0.997	0.998	4.62	15.786	0.953
2344	D:0.784	0.319	N:0.345	26.8	N:-0.81	P:0.990	B:0.435	N:0.001	0.999	1	4.27	8.312	0.852
1802	D:0.944	0.573	H:4.04	35	D:-4.53	D:1	D:0.998	D:0	1	1	4.26	13.182	0.852
2722	D:0.837	0.199	L:1.53	26	N:-0.99	P:0.933	P:0.467	D:0	0.996	0.991	2.03	11.835	0.953
1522	N/A	N/A	N/A	N/A	N/A	N/A	N/A	N/A	N/A	N/A	N/A	N/A	N/A

ALS: amyotrophic lateral sclerosis, ANXA11: Annexin A11, MND: motor neuron disease identification number, cDNA: complementary DNA. dbSNP147: Single Nucleotide Polymorphism Database 147. gnomAD: The Genome Aggregation Database. EAS: East Asian. SIFT: Sorting Intolerant From Tolerant. Pred: prediction. FATHMM: Functional Analysis through Hidden Markov Models. VEST3: Variant Effect Scoring Tool 3. CADD_phred: Combined Annotation Dependent Depletion_Phred. PP2_HDIV: Polymorphism Phenotyping v2_HumDiv. PP2_HVAR: Polymorphism Phenotyping v2_HumVar. LRT: Likelihood ratio test. DANN: Deleterious annotation of genetic variants using neural networks. PhastCons20way GERPrs: Genomic Evolutionary Rate Profiling rejected substitutions. SALS: sporadic amyotrophic lateral sclerosis. VUS: variants of uncertain significance. LPV: likely pathogenic variants. N/A: not available. D: deleterious or probably damaging. T: tolerable. M: medium. L: low. H: high. N: neutral. P: possibly damaging

Supplementary Table 2. Clinical characteristics of ALS patients with *ANXA11* variants

MND	Ethnicity	Protein	cDNA	Domain	Sex	Age of onset (years)	Early slope†	Late slope††	Site of onset	Phenotype[1]	Other neurological manifestations		Tracheostomy -free survival duration††††	Occurrence of endpoint (months, event type)
											Cognition	Others†††		
1876	Korean	p.P36R	NM_001157.2:c.107C>G	LCD	F	79	1.33	1.38	Bulbar	Bulbar	ALS-FTD(svPPA)	None	60	No
1742	Korean	p.P36R	NM_001157.2:c.107C>G	LCD	M	71	1.00	1.00	Limb	Classic	ALS-FTD(nfavPPA)	None	45	Yes (45, tracheostomy)
2011	Korean	p.P36R	NM_001157.2:c.107C>G	LCD	M	76	1.00	0.71	Bulbar	Bulbar	ALS-FTD(nfavPPA)	None	27	Yes (27, death)
2516	Korean	p.P36R	NM_001157.2:c.107C>G	LCD	F	73	1.11	1.11	Limb	Classic	ALSci	None	24	Yes (24, tracheostomy)
2807	Korean	p.P36R	NM_001157.2:c.107C>G	LCD	M	65	1.30	1.92	Bulbar	Bulbar	ALS-FTD(bvFTD)	None	22	No
2885	Korean	p.P36R	NM_001157.2:c.107C>G	LCD	F	83	1.88	1.83	Bulbar	Bulbar	ALSci	None	16	No
2925	Korean	p.P36R	NM_001157.2:c.107C>G	LCD	F	64	1.20	2.67	Limb	Classic	ALS-FTD(bvFTD)	None	16	No
1656	Korean	p.G38R	NM_001157.2:c.112G>A	LCD	F	66	2.40	3.14	Bulbar	Bulbar	N/A	None	57	Yes (57, permanent NIV)
2256	Korean	p.D40G	NM_001157.2:c.119A>G	LCD	F	54	6.00	2.29	Bulbar	Bulbar	ALS-FTD(bvFTD)	None	19	Yes (19, tracheostomy)
1818	Korean	p.D40G	NM_001157.2:c.119A>G	LCD	M	68	0.59	2.10	Bulbar	Bulbar	ALS-FTD(svPPA)	None	49	Yes (49, death)
2660	Korean	p.D40G	NM_001157.2:c.119A>G	LCD	M	75	2.00	2.00	Limb	Classic	ALSci	None	18	Yes (18, tracheostomy)
1761	Korean	p.G137R	NM_001157.2:c.409G>A	LCD	M	64	0.45	0.45	Limb	Flail arm	N/A	None	58	Yes (58, death)
2303	Korean	p.P185Qfs*29	NM_001157.2:c.554del	LCD	M	63	0.58	0.58	Limb	Classic	ALSci	None	30	Yes (30, tracheostomy)
861	Korean	p.G228Lfs*29	NM_001157.2:c.682_686delins TTG	Annexin 1	M	50	0.86	1.00	Limb	Classic	ALSci	None	38	Yes (38, tracheostomy)
1943	Korean	p.D277A	NM_001157.2:c.830A>C	intron 2	F	54	0.32	0.25	Limb	Classic	ALSci	None	85	No
2474	Korean	p.R302C	NM_001157.2:c.904C>T	Annexin 2	M	66	0.80	1.33	Bulbar	Bulbar	N/A	None	17	Yes (17, tracheostomy)
2350	Korean	p.R336W	NM_001157.2:c.1006C>T	Annexin 2	M	63	0.48	0.09	Limb	Classic	ALS-pure	None	34	Yes (34, tracheostomy)
1653	Korean	p.H390P	NM_001157.2:c.1169A>C	Annexin 3	F	56	2.20	3.00	Bulbar	Bulbar	ALS-pure	None	18	Yes (18, tracheostomy)
2103	Korean	p.H390P	NM_001157.2:c.1169A>C	Annexin 3	F	50	0.50	0.42	Limb	Classic	ALSci	None	33	No
2437	Korean	p.H390P	NM_001157.2:c.1169A>C	Annexin 3	M	76	2.00	2.00	Limb	Pyramidal	ALS-pure	None	15	Yes (15, death)
2518	Korean	p.Q399P	NM_001157.2:c.1196A>C	Annexin 3	M	56	1.00	1.00	Limb	Classic	ALS-pure	None	15	No
2725	Korean	p.A434D	NM_001157.2:c.1301C>A	intron 4	M	66	0.61	2.63	Bulbar	Bulbar	ALSci	None	27	Yes (27, tracheostomy)
2344	Korean	p.R452Q	NM_001157.2:c.1355G>A	Annexin 4	M	54	0.90	0.89	Limb	Classic	ALS-pure	None	42	No
1802	Korean	p.R456H	NM_001157.2:c.1367G>A	Annexin 4	M	56	0.77	1.10	Bulbar	Bulbar	ALSci	None	29	Yes (29, tracheostomy)
2722	Korean	p.H483Y	NM_001157.2:c.1447C>T	Annexin 4	F	64	0.26	0.22	Limb	Flail leg	ALSci	None	60	No
1522	Korean	-	NM_001157.2:c.1458+7G>A	Annexin 4	F	56	0.20	0.83	Limb	Classic	ALS-pure	None	35	No

† Early slope = (48 - ALSFRS-R at first consultation)/months from symptom onset to the first consultation
†† Late slope = (ALSFRS-R at first consultation – ALSFRS-R score at follow-up period)/months passed since the first visit
††† Others include inclusion body myositis and Paget's disease of bone which are pleiotropic degenerative diseases characterized as multisystem proteinopathies.
†††† The time from the onset of symptoms to the endpoint or the censoring date of May 31, 2021, was used to determine the patients' tracheostomy-free survival duration. The endpoint is defined as the occurrence of death, tracheostomy, and permanent noninvasive positive pressure ventilation (>22 hours daily for more than seven days).
ALS: amyotrophic lateral sclerosis, ANXA11: Annexin A11, MND: Motor neuron disease identification number, LCD: Low-complexity domain. M: male. F: female. ALS-FTD: amyotrophic lateral sclerosis-frontotemporal dementia. ALSci: amyotrophic lateral sclerosis with cognitive impairment. ALS-pure: amyotrophic lateral sclerosis-pure(without cognitive impairment). svPPA: semantic variant primary progressive aphasia. nfavPPA: nonfluent/agrammatic variant. bvFTD: behavioral variant frontotemporal dementia. NIV: noninvasive positive pressure ventilation N/A: not available.

Supplementary Table 3 Demographic and clinical features of subgroups of patients with *ANXA11* variants classified according to variant location, ALS patients with *ANXA11* variants, and ALS patients in Korea[2,3,4]

	**LCD variant group (n=13)**	**ANX domain variant group (n=13)**	**ALS patients with *ANXA11* variants**	**ALS patients in Korea**
Male:female ratio	1.17	1.60	1.36	1.60
Age of onset<br>Mean±SD	69.3±7.8	59.0±7.5	64.2±9.2	60.4±12.4
Early slope†<br>Median (range)	1.20 (1.00-1.88)	0.77 (0.32-0.90)	0.95 (0.20-1.32)	0.60 (0.33-1.00)††
Site of onset<br>Bulbar:spinal ratio	1.17	0.44	0.73	0.20
Presence of FTD, %	53.8	0	26.9	4.8
Survival duration, months	45.0††† (24.0-57.0)	34.0††† (27.0-38.0)	38.0††† (24.0-58.0)	50.0††††
Presence of fALS, %	0	0	0	2.0~3.1

† Early slope = (48 - ALSFRS-R at first consultation)/months from symptom onset to the first consultation
†† Data were extracted from 882 ALS patients who underwent next-generation sequencing recruited from the ALS clinic of Hanyang University Hospital in Seoul, Korea.
††† The figures were expressed as the median and interquartile range
†††† Data were presented as the mean survival period
ALS: amyotrophic lateral sclerosis, ANXA11: Annexin A11, LCD: Low-Complexity Domain. ANX: Annexin. ALS: amyotrophic lateral sclerosis. SD: Standard deviation. FTD: Frontotemporal dementia. fALS: familial amyotrophic lateral sclerosis.

Supplementary Figure 1

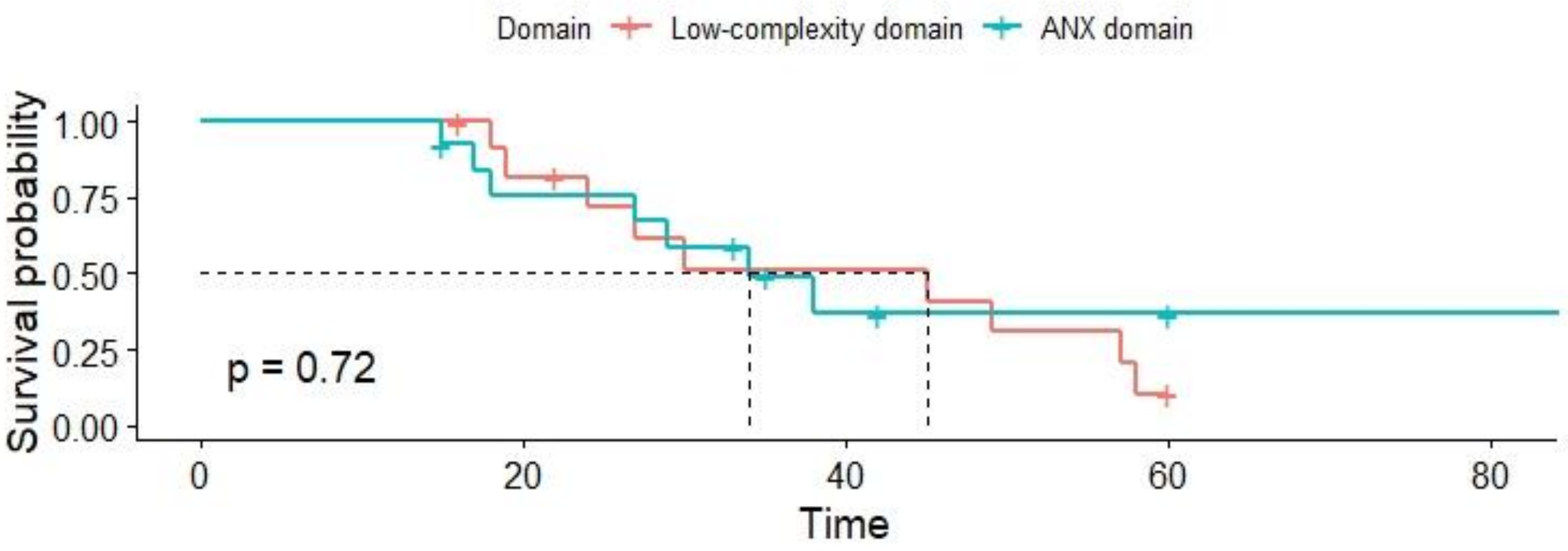

Supplementary Figure 1 Kaplan-Meier survival curves. The survival rate did not show significant difference between the LCD variant group and ANX domain variant group.

Supplementary Figure 2

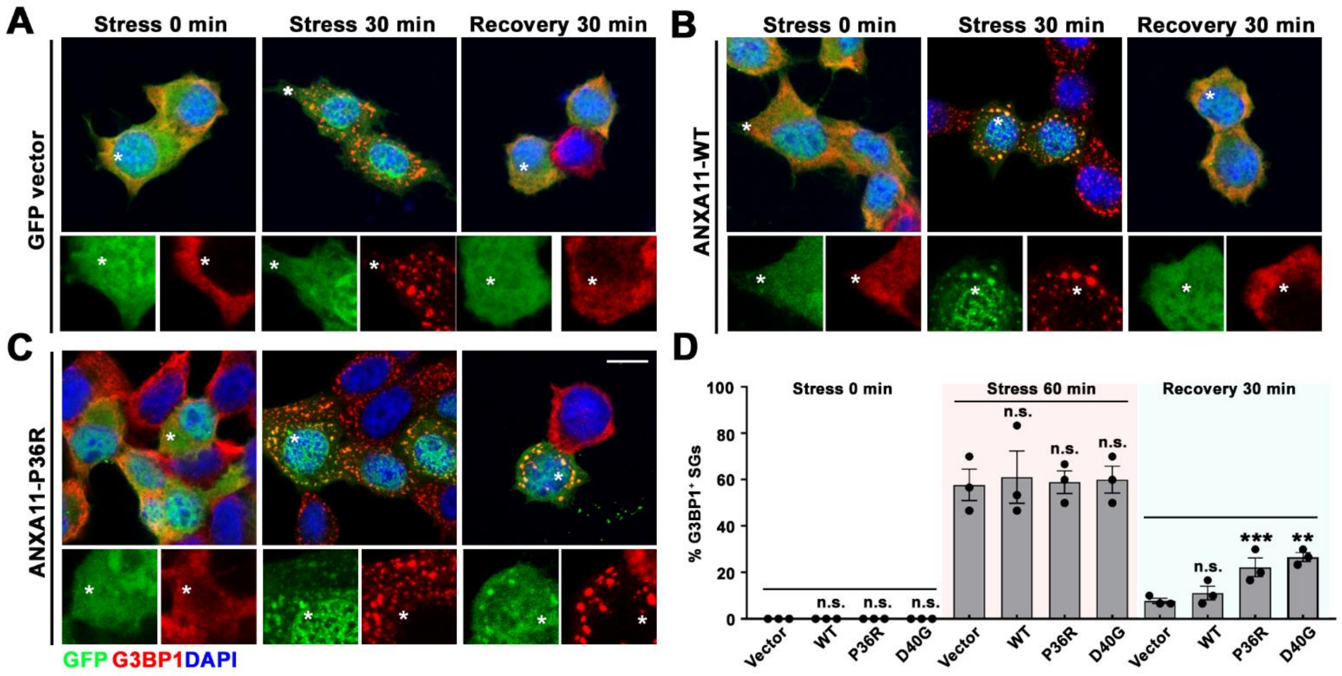

Supplementary Figure 2 *ANXA11* variants affect stress granule disassembly. (A-C) WT or mutant ANXA11-GFP was transfected in NSC-34 cells (Stress 0 min) and treated with 0.5 mM sodium arsenite for 30 min (Stress 30 min) followed by recovery incubation with fresh medium for 30 min (Recovery 30 min). A GFP vector used as a control. SGs were visualized by immunofluorescence using anti-G3BP1 antibody. DAPI was used to detect nuclei. The bottom panels show higher magnification views of the regions indicated by white asterisk. The scale bar represents 10 μm. (D) The percentage of cells containing G3BP1-positive SGs in transfected cells (30 cells, n =3) during the assembly and disassembly phases was determined and shown in the graph. The values are the mean ± S.E.M. All comparisons were made against GFP vector. **$p < 0.01$, ***$p < 0.05$, n.s. not significant; one-way ANOVA with post-hoc Tukey tests.

References

1. Chiò A, Calvo A, Moglia C, Mazzini L, Mora G. Phenotypic heterogeneity of amyotrophic lateral sclerosis: a population based study. Journal of Neurology, Neurosurgery & Psychiatry. 2011;82(7):740-746.
2. Jun KY, Park J, Oh K-W, et al. Epidemiology of ALS in Korea using nationwide big data. Journal of Neurology, Neurosurgery & Psychiatry. 2019;90(4):395-403.
3. Oh S-I, Park A, Kim H-J, et al. Spectrum of Cognitive Impairment in Korean ALS Patients without Known Genetic Mutations. PLoS ONE. 2014;9(2):e87163.
4. Bae JS, Hong Y-H, Baek W, et al. Current status of the diagnosis and management of amyotrophic lateral sclerosis in Korea: a multi-center cross-sectional study. Journal of Clinical Neurology. 2012;8(4):293-300.
